# Construction of CuS/Au Heterostructure through a Simple Photoreduction Route for Enhanced Electrochemical Hydrogen Evolution and Photocatalysis

**DOI:** 10.1038/srep34738

**Published:** 2016-10-05

**Authors:** Mrinmoyee Basu, Roshan Nazir, Pragati Fageria, Surojit Pande

**Affiliations:** 1 Department of Chemistry, BITS Pilani, Pilani, Rajasthan, 333031, India

## Abstract

An efficient Hydrogen evolution catalyst has been developed by decorating Au nanoparticle on the surface of CuS nanostructure following a green and environmental friendly approach. CuS nanostructure is synthesized through a simple wet-chemical route. CuS being a visible light photocatalyst is introduced to function as an efficient reducing agent. Photogenerated electron is used to reduce Au(III) on the surface of CuS to prepare CuS/Au heterostructure. The as-obtained heterostructure shows excellent performance in electrochemical H_2_ evolution reaction with promising durability in acidic condition, which could work as an efficient alternative for novel metals. The most efficient CuS-Au heterostructure can generate 10 mA/cm^2^ current density upon application of 0.179 V vs. RHE. CuS-Au heterostructure can also perform as an efficient photocatalyst for the degradation of organic pollutant. This dual nature of CuS and CuS/Au both in electrocatalysis and photocatalysis has been unveiled in this study.

Hydrogen is being considered as the surrogate for the limited fossil fuels hidden in the earth crust to address the global energy demand^1^. Hydrogen can be generated from different sources like water, coal, biomass etc. Generation of hydrogen from water splitting can provide the unique green path way without having any environmental pollution. Plenty of research have already been dedicated to discover efficient catalyst, which can drive the hydrogen evolution reaction (HER) with application of a minimum over potential^2^,^3^,^4^,^5^,^6^,^7^. The most effective and efficient catalyst for HER is Pt and Pt based compounds, which can generate hydrogen almost having onset potential 0 V 8. The main drawback is the cost and the availability of Pt, which restricts the industrial application. Therefore, to establish hydrogen as the competitive energy source, it is very important to develop low cost and efficient catalyst, which can generate large scale of hydrogen in very low over potential.

To replace Pt different earth-abundant metal and their corresponding compounds are also studied as catalyst for HER. Transition-metal chalcogenides are possessing similar electronic structure like noble metals, being studied as HER catalyst from last decade and establish themselves as alternative to Pt^9^. Mostly used non-precious-metal as an alternative to Pt are MoS_2_, MoC_2_, WS_2_, WSe_2_, CoS_2_, CoSe_2_, NiS_2_, FeS_2_, Ni_2_P etc^10^,^11^,^12^,^13^,^14^,^15^,^16^,^17^,^18^. Different metal alloys like Ni-Mo, Ni-Fe, Ni-Mo-Zn are also successfully used as catalyst for HER reaction^19^,^20^,^21^. Recently CuS is being studied for its electrocatalytic activity in HER reaction along with its photocatalytic performance^22^. It is already proved both experimentally and theoretically that the sulfur atoms on the exposed surface of transition metal chalcogenides increases the HER activity of a material^23^. At the exposed surface sulfur atoms are much more unsaturated which plays an important role to increase the electrocatalytic activity of MoS_2_^24^. CuS posses layered crystal structure having weak van der Waals interactions between two double layers of Cu_2_S_2_^25^. Sulfur present in the CuS have empty 3-p orbitals which further helps to easy capture electrons and promotes electron transportation. Although CuS is chosen very rarely as a suitable electrocatalyst for HER reaction, but the prompt feasibility of electron acceptance and transportation by CuS can establish it as a promising HER catalyst. Recently Xi *et al.*, reported electrocatalytic activity of CuS and NiCo_2_O_4_/CuS heterostructure^26^.

Being inspired by this report we have also synthesized CuS following a simple wet-chemical route. CuS is a widely studied efficient visible light active photocatalyst and also very active under indoor light. In this study we have introduced a photochemical reduction approach to synthesize Au nanoparticle on the surface of CuS. Initially, CuS was excited under visible light to generate electrons and holes and simultaneously HAuCl_4_ undergoes photoreduction by photo-generated electrons. Following this green technique without using any reducing agent we have synthesized Au nanoparticle on the surface of CuS (CuS-Au-n, with varying Au loading). Finally, the synthesized CuS and CuS-Au-n were studied as catalyst in electrocatalytic HER reaction and also in photocatalysis (Fig. 1). Best catalytic efficiency is achieved in case of CuS-Au-3 (weight percentage of Au = 19.98) and it can generate 10 mA/cm^2^ current density in −0.179 V vs. RHE. CuS-Au-3 also shows the best performance in photocatalysis with rate constant (k) value 3.7 × 10^−1^ min^−1^ which is 21 times higher compared to bare CuS. Superior electrocatalytic and photocatalytic activity of CuS-Au-3 is explained with the help of impedance spectroscopy and photoluminescence study, respectively.

## Results and Discussion

**Figure 2:**
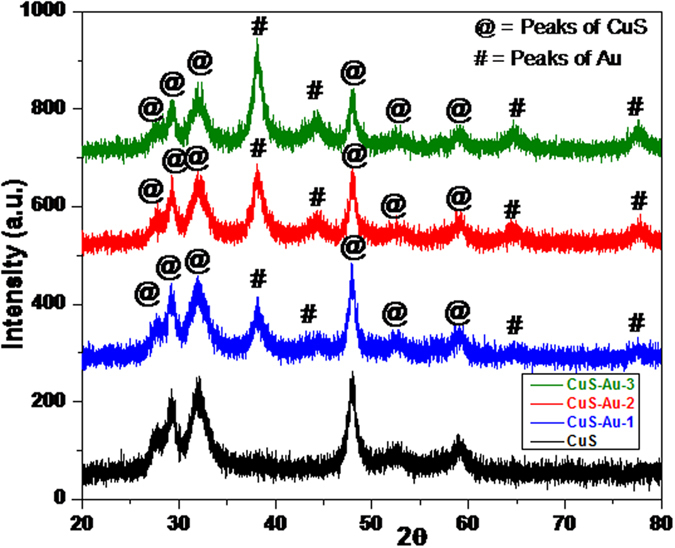
PXRD pattern of CuS and CuS-Au-n (n = 1, 2, 3) showing the variation in the intensities of the highest intense peak of CuS and Au.

With the help of powder X-ray diffraction analysis phase purity and crystallinity of the synthesized CuS and CuS-Au-n (n = 1, 2, 3, 4) samples were determined. Figure 2 shows the PXRD pattern of bare CuS and CuS-Au-n (n = 1, 2, 3) samples. XRD of CuS is well matched with the JCPDS no 00-006-0464. In the present system CuS crystallizes in hexagonal crystal system. Presence of any impurity, intermediate product, starting compound is not detected through XRD analysis. XRD pattern of CuS-Au-n clearly shows three diffraction of (111), (200) & (220) planes which are the characteristic peaks due to Au and well matched with the JCPDS no 04-0784. XRD pattern of Au nanoparticle suggests that Au nanoparticle crystallizes in cubic system. With the increase in concentration of HAuCl_4_, it was observed that there is successive enhancement in the intensity of the corresponding peak of Au on CuS. Finally, in case of CuS-Au-3 it is very clear that peaks of Au is dominating over the peaks of CuS. From the comparison of the highest intense peaks of CuS and Au it is clear that in case of CuS-Au-1, CuS-Au-2, CuS-Au-3 CuS:Au peak intensity ratio is ~ 2:1, ~3:2, ~1:1, respectively. Therefore, from this observation it can be claimed that surface of CuS in CuS-Au-3 sample mostly covered with Au nanoparticle. Excess loading of Au on CuS can also be claimed from XRD pattern of CuS-Au-4 (SI, Figure S1).

**Figure 3:**
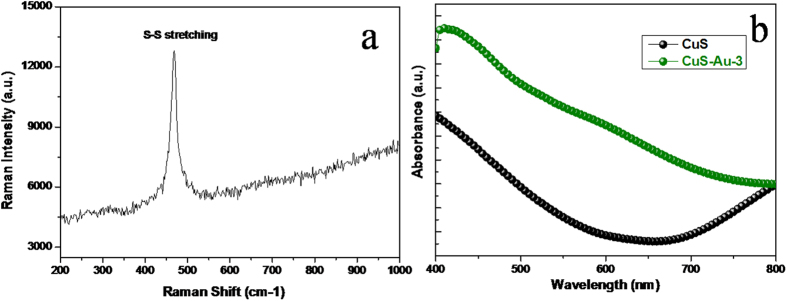
(**a**) Raman spectrum of CuS only and (**b**) UV-vis absorption spectrum of CuS and CuS-Au-n (n = 3 only).

Crytallinity of CuS was determined with the help of Raman spectroscopy, which is shown in Fig. 3a. It exhibits a very strong peak at 468 cm^−1^ which is due to the vibration mode of S-S covalent bonds in CuS. Peak at 468 cm^−1^ is corresponding to the well-known A1g mode of CuS^27^. It is worth mentioning that there is no additional impurity peaks are present for any copper substrate like Cu(OH)_2_ and also un-reacted precursor compound.

Light absorption ability of CuS nanostructure and CuS/Au heterostructure was determined by UV-vis spectroscopy utilizing the ethanolic dispersion of the respective materials and shown in Fig. 3b. Both the spectra show an enhanced absorption closer to the near IR region. Literature reports that covellite CuS shows two characteristic absorption peaks: one in visible region and another in near IR region^28^. In our present case, CuS exhibits enhanced absorption near 400 nm having the band edge at 550 nm, which again dictates about the band gap about 2.2 eV. Due to having absorption both in visible and in near IR region a valley is visualized in between 500 to 780 nm and this phenomena is supported in literature also^28^. However, after deposition of Au nanoparticle on the surface of CuS, there is a certain increase in the absorption intensity in the visible region, which further wraps the valley of CuS. The absorption localized in the region 500 to 600 nm is probably due to the surface Plasmon resonance (SPR) effect of Au nanoparticles. Therefore, Au nanoparticle present on the surface of CuS results in the enhanced light absorption in the visible region.

To determine the surface electronic state and the composition of the product XPS analysis was carried out. Wide scan spectra of CuS sample indicates the presence of Cu and S as well as C and O due to the exposure in air, shown in SI, Figure S2a. XPS spectrum of CuS reveals that the Cu2p_3/2_ peak has binding energy at 231.8 eV which is the characteristic of CuS and this value is 0.7 eV lower than that of Cu_2_S^29^,^30^. Binding energies of Cu2p_1/2_ and Cu2p_3/2_ peaks at 951.7 and 931.8 eV are shown in SI, Figure S2b. Two satellite peaks centered at 943.1 and 963.5 eV indicates the presence of the paramagnetic chemical state of Cu^2+^. Binding energy of Cu2p_3/2_ and Cu2p_1/2_ are separated by 20 eV are essentially identical binding energies of Cu2p of Cu(II)^31^. The corresponding XPS spectra of S have been shown in SI, Figure S2c. The binding energy peaks observed in the S2p spectrum at 161.7 and 162.7 eV, which are attributed to the S2p_3/2_ and S2p_1/2_ states respectively, also confirm the formation of CuS. A wide scan spectrum of CuS-Au-3 sample shows the presence of Au, S, Cu, O and C (SI, Figure S3a). Binding energy of Cu and S remains unchanged even after deposition of Au on the surface of CuS (SI, Figure S3b,c). The binding energy of Au 4f is found as Au4f_7/2_ and Au4f_5/2_ at 83.7 and 87.4 eV respectively (SI, Figure S3d). This binding energy values are consistent with the bulk gold and indicates the formation of Au(0) on the surface of CuS^32^,^33^,^34^.

**Figure 4:**
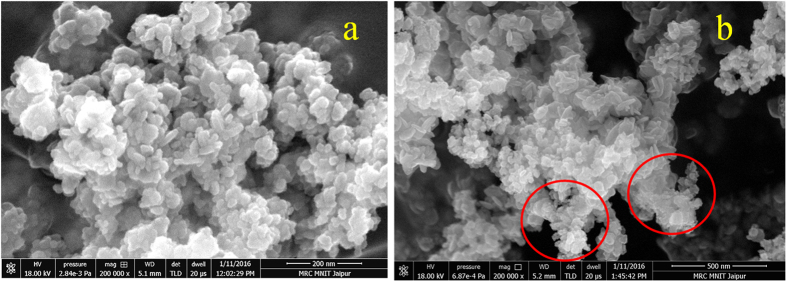
FESEM image of (**a**) CuS stacked plates and (**b**) CuS-Au-3 on CuS surface. Encircled area shows attachment of small particles on CuS plates.

To observe the possible growth of CuS and successive deposition of Au on the surface of CuS, we have carried out field emission scanning electron microscopy (FESEM) measurement for both CuS and CuS-Au-n (n = 3) (Fig. 4a,b). It can be seen from Fig. 4a that highly dense and small nanoplates of CuS are present. Nanoplates are having the edge length ~25 nm. SEM-EDS analysis of CuS shows the presence of Cu and S having atomic % ratio ~1:1, which again confirms the formation of CuS (SI, Figure S5). After photochemical deposition of Au on the surface of CuS, small particles of Au were observed through FESEM analysis, highlighted by encircling in Fig. 4b. With the increase in HAuCl_4_ concentration there is successive increase in the density of Au nanoparticle on the surface of CuS (SI, Figure S4). SEM-EDS analysis clearly shows the presence of Cu, S, Au in CuS-Au-n (n = 1, 2, 3) samples (SI, Figure S6). It is very hard to determine the particle size of Au from SEM analysis due to its small particle size. SEM-EDS mapping of CuS-Au-3 also confirms the presence of Cu and S and uniform distribution of Au on CuS nanostructure (SI, Figure S7). ICP-OES analysis was carried out to determine the exact loading of Au on CuS in case of CuS-Au-n (n = 1, 2, 3) and the data is presented in SI, Table S1. From ICP-OES data it is clear that with increase in the amount of HAuCl_4_ in the photo reduction process; there is successive increase of Au loading on CuS. Maximum Au loading is observed in CuS-Au-3 (weight % = 19.98) followed by CuS-Au-2 (weight % = 12.85) and CuS-Au-1 (weight % = 5.92).

**Figure 5:**
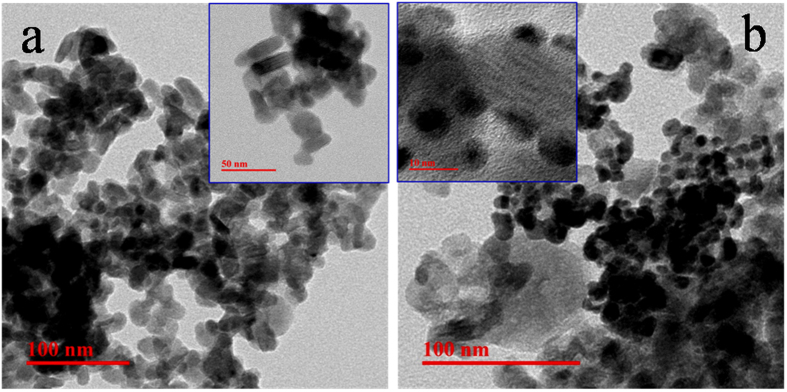
Low magnification TEM image of CuS (**a**) and CuS-Au-3 (**b**). Inset shows high magnification images of very small plates of CuS and very small particles of Au on CuS surface.

**Figure 6:**
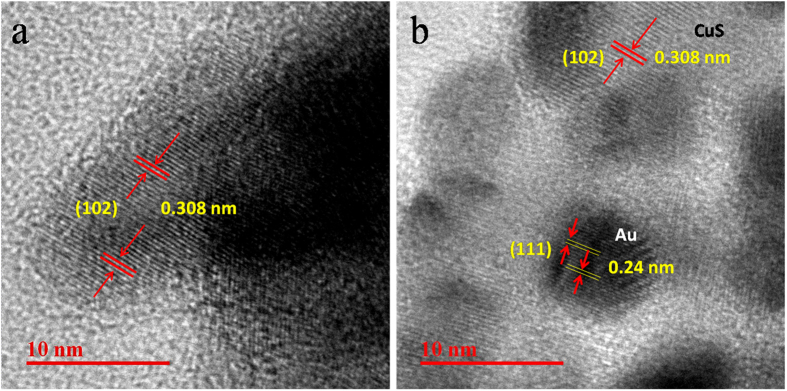
HRTEM image of (**a**) CuS and (**b**) CuS-Au-3.

With the help of transmission electron microscopy (TEM) analysis morphology of CuS was further verified. Figure 5a shows the typical TEM images of CuS. From low magnification TEM image it is clear that highly dense CuS nanoplates, which are not properly aligned, are synthesized following our method. High magnification image shows that there are some light shaded small plates and also some dark strips (inset of Fig. 5a). Dark strips are corresponding to the CuS plates, which are present as perpendicular to the basal plane. Both the alignment (perpendicular and parallel) are observed from the high magnification image. Edge lengths of the CuS plates are ~25 nm, which is in agreement with SEM images. TEM-EDS mapping of CuS shows the presence of uniform distribution of Cu and S as elements (SI, Figure S8). To determine the crystallinity of the as-synthesized product HRTEM was carried out. HRTEM image clearly demonstrate the lattice spacing of the crystalline CuS is 0.308 nm, corresponding to the spacing between two crystal plane (102) (Fig. 6a) which matches well with the literature^35^,^36^. Decoration of photo chemically deposited Au nanoparticle on the plates of CuS was also verified with the help of TEM analysis. Figure 5b shows the arrangement of Au nanoparticle on the surface of CuS plates. High magnification image shown in the inset of Fig. 5b confirms that the Au nanoparticles deposited on CuS have particle size ~5–10 nm. HRTEM image was taken on the surface of Au deposited CuS plates which clearly shows the interface between Au nanoparticles and CuS nanoplates. Figure 6b shows the ‘d’ spacing calculated from the lattice spacing of deposited Au nanoparticle is ~0.24 nm which is due to the spacing between two crystal planes (111) of Au and it is well matched with the literature^34^.

## Electrocatalytic Activity

**Figure 7:**
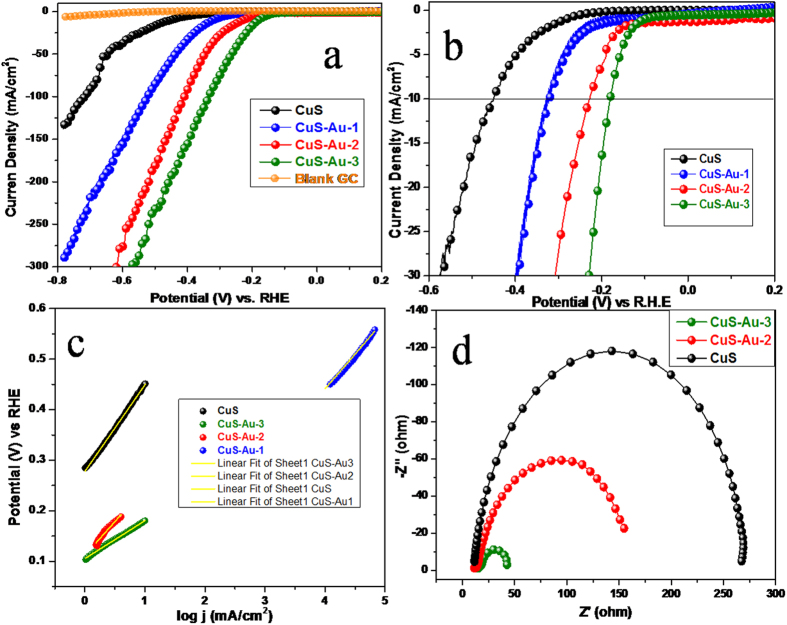
(**a**,**b**) Polarization curves for bare GC, CuS, CuS-Au-1, CuS-Au-2 and CuS-Au-3 in 0.5 M H_2_SO_4_. (**c**) Tafel plots of CuS, CuS-Au-1, CuS-Au-2, and CuS-Au-3, (**d**) Nyquist plot of CuS, CuS-Au-2 and CuS-Au-3.

The electrocatalytic activity of CuS and CuS-Au-n was evaluated by using linear sweep voltammogram (LSV) technique. All the electrochemical measurements for hydrogen evolution were carried out in 0.5 M H_2_SO_4_ aqueous solution with scan rate of 50 mV/s. Potentials are recorded with reference to Ag/AgCl and reported with respect to reversible hydrogen electrode and the polarization curves are shown in Fig. 7a,b. Au nanoparticle decorated CuS shows a remarkably enhanced current density with successive anodic shift in the onset potential compared to bare CuS. With successful increase in the amount of Au on the surface of CuS, there is successive enhancement in the current density and at the same time there is clear anodic shift in the onset potential. CuS nanoplates require −0.449 V potential to generate current density of 10 mA/cm^2^. In comparison, to achieve current density of 10 mA/cm^2^, CuS-Au-1 needs overpotential −0.324 V. Again, in case of CuS-Au-2 and CuS-Au-3, it needs −0.226 V and −0.179 V, respectively to reach current density of 10 mA/cm^2^. CuS-Au-3 shows best catalytic activity but still it is less compared to Pt, as reported in the literature^37^. In all cases, Au nanoparticle modified CuS shows the enhanced catalytic activity compared to the bare CuS. At potential −0.475 V bare CuS can generate only 12.5 mA/cm^2^ current density whereas, in case of CuS-Au-1 the value increased up to 70.63 mA/cm^2^. Current density further increased for CuS-Au-2 to 159.6 mA/cm^2^ and the highest current density was achieved in case of CuS-Au-3, which is 215.5 mA/cm^2^. This superior activity of CuS-Au compared to CuS may be attributed to the fact that Au nanoparticle on CuS surface can function as an electron sink and successfully snatch the carrier from the surface of CuS and transport to electrolyte where it reacts with H^+^ ion to generate H_2_. For better understanding about the contribution on Au nanoparticle on the surface of CuS, electrocatalytic activity of CuS-Au-4 was also checked. With further increase in the concentration of Au certainly decrease the electrocatalytic activity of CuS and successive cathodic shift in the onset potential were observed (SI, Figure S9). In presence of Au nanoparticle initially a positive shift in the onset potential was observed where Au nanoparticle functions as electron collectors and makes faster electron transportation. But excess amount of Au on CuS further decreases the electrocatalytic activity. The possible reasons for this decrease in the electrocatalytic activity are firstly, high amount of Au loading may cover all the active sites of CuS and further hindering its contact with water molecule. Secondly, more amount of Au may work as aggregates and further large particle size leads to disappearance of surface effects. Electrocataytic activity of pure Au nanoparticle was also checked and shown SI, Figure S10. Pure Au nanoparticle needs −0.531 V vs. RHE to generate 10 mA/cm^2^ current density. It can achieve only 16.8 mA/cm^2^ current density upon application of −0.58 V vs. RHE, which is poor as compared to CuS and CuS-Au-3.

Tafel slope is the intrinsic property of a material and focus the rate determining step in HER reaction. Tafel slope is also useful to determine the effectiveness of a catalyst. The linear portion of the Tafel plots were fitted in the Tafel equation (η = b log(j) + a, where b is the Tafel slope)^38^,^39^. The values of Tafel slope for CuS, CuS-Au-1, CuS-Au-2 and CuS-Au-3 are 171, 138, 133 and 75 mV/decade, respectively (Fig. 7c). Tafel slope data indicates that with increase in the amount of Au nanoparticle on CuS, there is further enhancement in the electrocatalytic activity of CuS. Tafel slop value also confirms that among all these catalysts, CuS-Au-3 is electrocatalytically much more active than others. Generally HER reaction follows two possible mechanisms in acidic medium: It may be Volmer-tafel or it may be Volmer-Heyrovsky. The First step (equation 1) is the Volmer step also called discharge step:


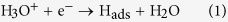



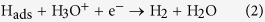



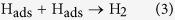


This reaction is either followed by Heyrovsky process or called as desorption step (equation 2) or Tafel step which is also called recombination step (equation 3). It is expected that the Tafel slope should be 120, 40 or 30 mV/decade if the Volmer, Heyrovsky and Tafel is the respective rate determining step. In case of CuS, HER reaction follows Volmer-Heyrovsky mechanism following Volmer as the rate determining step. In case CuS-Au-3 the HER reaction follows same mechanism but the rate determining step is the Heyrovsky reaction. Stability of CuS and CuS-Au-3 were checked upon continuous scan of 1000 cycles. It is very clear from SI, Figure S11, there is negligible change in the current density and onset potential as well, which further claims the stability of CuS and CuS-Au-3 in acidic medium. Digital image of H_2_ evolution from GC electrode is shown in SI, Figure S12.

## Impedance Measurement

To have a clear understanding about the electrocatalytic activity of CuS and CuS-Au-n sample, we have carried out electrochemical impedance measurement. Electrochemical Impedance study clearly gives the idea about the ease of electron transportation on different electrode surface. Nyquist impedance plots have been measured for CuS at −0.368 V vs. RHE, which is the onset potential for CuS. On the other hand for CuS-Au-2 and CuS-Au-3, it was measured at the onset potentials of the respective compounds. The evaluated data can be fitted with an equivalent circuit composed of a constant phase element (CPE) for CuS and CuS-Au-n and the resistance (R_S_), which represent the core resistance of the material and R_ct_ dictates about the charge transfer resistance from the electrode surface to electrolyte.

Impedance curve has been fitted and the resistance values are summarized in Table 1 (Fig. 7d). Rs resistance of CuS and, CuS-Au-2 and CuS-Au-3 are 10.5, 11.7 and 14.8 Ω, respectively. With the increase in the amount of Au on CuS surface it is very clear that R_ct_ value decreases from CuS to CuS-Au-2 and that to CuS-Au-3. The R_ct_ value decreases very sharply from CuS to CuS-Au-2 to finally CuS-Au-3. All these data have been noted in table no 1. The charge transfer resistance from CuS to electrolyte is 255.4 Ω in case of CuS. Whereas, in case of CuS-Au-2 this value decreases to 143.7 Ω and finally for CuS-Au-3 it further decrease to 31.1 Ω. Successive decrease in the charge transfer resistance from CuS surface to electrolyte with the increase in the amount of deposited Au further depicts that Au helps in the faster charge transfer from electrode to electrolyte. Photochemically deposited Au nanoparticle on CuS surface can function as an electron sink^40^ or trap which captures the electrons from CuS upon application of external bias. The impedance results are in well agreement with our as-observed electrocatalysis result. Higher loading of Au nanoparticle on CuS surface further decreases the electrocatalytic activity. For comparison impedance spectra of CuS, CuS-Au-2, CuS-Au-3 and CuS-Au-4 is given in SI, Figure S13.

## PL Study

In case of semiconductor nanostructure, PL is an efficient and suitable tool to study the electron transfer, migration and at the same time the fate of the photogenerated electron hole-pair. Mechanism of electron transfer was determined with the help of photoluminescence (PL). PL study was carried out using the ethanolic dispersion of CuS at room temperature with an excitation wavelength of 350 nm (3.54 eV). In case of bare CuS a broad emission band in the visible region was observed centered at 509 nm (2.43 eV) (SI, Figure S14). This observation clearly matches with the reported literature^41^. Furthermore to understand the charge transfer process; PL study was also carried out for CuS-Au-3 sample at room temperature. CuS-Au-3 sample almost shows the same emission band with much lower band intensity. Originally, PL emission spectra result from the recombination of the free electrons and holes. Successive decrease in the emission intensity was observed in case of CuS-Au-3 compared to CuS, which may be attributed due to the electron sink nature of Au attached with CuS. Au decorated on CuS snatches the photogenerated electron from CuS, helps in inhibiting the recombination of charge carrier in CuS, which further results in lowered PL intensity.

### Photocatalysis

**Figure 8:**
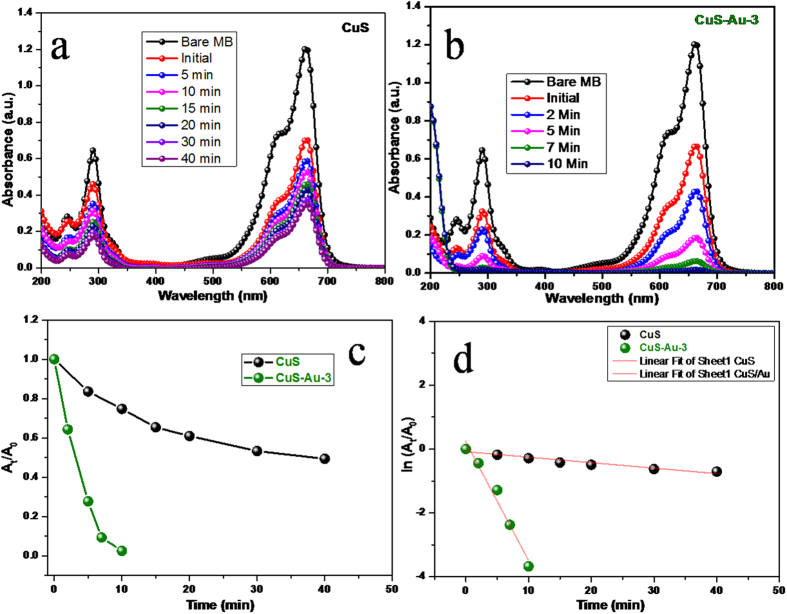
Photocatalytic decomposition of MB dye by using (**a**) CuS (**b**) CuS-Au-3 catalyst: absorbance vs. wavelength plot, (**c**) Plot of (A_t_/A_0_) vs. time for both CuS and CuS-Au-3, and (**d**) Plot of ln(A_t_/A_0_) vs. time for both CuS and CuS-Au-3. Conditions: [MB] = 2 × 10^−5^ M and catalyst = 10 mg.

To study the photocatalytic activity of CuS and CuS-Au-3, methylene blue (MB) was chosen as a model cationic dye. MB has optical absorbance maxima at 663 nm. Photocatalytic activity of CuS and CuS-Au-3 was estimated by scrutinizing consecutive decrease in the absorption intensity of MB with time under irradiation of visible light (Fig. 8a,b). Previously, we have unveiled that CuS hexagonal plates can function as photocatalyst under indoor light^22^. Here, CuS have the negative surface charge as it was synthesized in alkaline condition. So, cationic dye MB is easily adsorbed on the surface of CuS. MB does not degrade under visible light irradiation in absence of any catalyst. Under stirring in dark 41.6% MB was adsorbed on the surface of CuS whereas 44.6% on the surface of CuS-Au-3. CuS shows only 25% dye degradation within 10 min of reaction. On the other hand CuS-Au-3 shows enhanced photocatalytic activity with 97% dye degradation within 10 min (Fig. 8b). Comparative % dye removal efficiency of CuS and CuS-Au-3 is shown in SI, Figure S15. Plot of A_t_/A_0_ vs. t shows that the photocatalytic degradation follows pseudo-first order reaction in both the cases (Fig. 8c). From the plot of ln (A_t_/A_0_) vs. t, rate constant ‘k’ was determined. In case of bare CuS the value of ‘k’ is 1.7 × 10^−2^ min^−1^, whereas, when CuS-Au-3 was used as photocatalyst the value of ‘k’ increased 21 times and the value become 3.7 × 10^−1 ^min^−1^ (Fig. 8d). With the increase of MB concentration, degradation efficiency decreases. Photocatalytic performance of CuS-Au-3 sample was checked introducing MB concentration as 2.5 × 10^−5^ M. Keeping all other reaction parameters unaltered when the concentration of MB is increased there is little decrease in the catalytic efficiency still the reaction follows pseudo-first order kinetics as A_t_/A_0_ vs. t plot shows the exponential decay (SI, Figure S16). From the plot of ln(A_t_/A_0_) vs. t, ‘k’ is determined and the value is 1.9 × 10^−1^ min^−1^ which is ~0.5 times lowered compared to the previous concentration.

**Figure 9:**
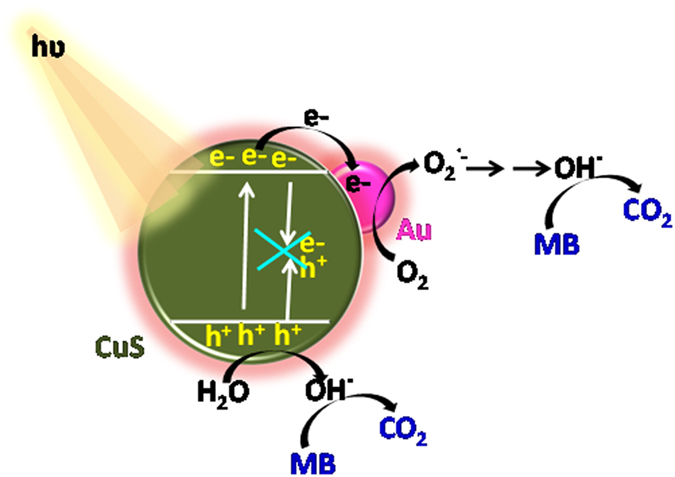
Schematic representation of MB degradation under visible light irradiation on CuS decorated with Au nanoparticles.

From UV-vis absorption of CuS-Au it was observed that Au nanoparticles enhance visible light absorbance of CuS nanoplates. PL study also clearly dictates that Au nanoparticle snatches the photogenerated electrons from the surface of CuS and readily transfers to MB which further results in high degradation efficiency with enhanced ‘k’ value. Au nanoparticle present on the surface of CuS nanoplates executes duel property to enhance the activity of CuS: It enhances the visible light absorption and at the same time it delays the recombination of photogenerated electron hole pair. CuS-Au-3 shows better performance in photocatalysis than CuS, which can be explained by the work function values. Reported work function value for Au and CuS is 5.1 eV and 4.95 eV, respectively^34^,^42^. So, in CuS-Au-3, a Schottky barrier will form, which helps in electron transfer between lower work function (CuS) material to higher (Au) work function material^43^,^44^,^45^,^46^. Upon irradiation with visible light, electrons will be excited to the conduction band (CB) leaving behind holes in valence band (VB) of CuS. Now, the excited electron can easily be shifted from the CB of CuS to the Au nanoparticle surface due to higher work function. These electrons react with dissolved oxygen and helps in the formation of superoxide radical anion (O_2_^˙−^), which also react with H_2_O to from hydroxyl radicals (OH˙)^40^. On the other hand holes present in the VB of CuS also reacts with H_2_O to generate (OH˙). These hydroxyl radicals are main active species for MB dye degradation. Au metal nanoparticles on CuS surface behave as an electron sink or trap for photogenerated electrons and can easily adsorb electrons from the CB of CuS and prevent immediate recombination^40^,^47^,^48^. Hence, CuS-Au-3 exhibit better photocatalytic efficiency shows ~97% dye degradation with in 10 min which is much faster compared to CuS. Mechanism of the photocatalysis is shown in Fig. 9.

In summary, the Au nanoparticle decorated CuS nanoplates have been fabricated via photochemical reduction of Au ions to Au nanoparticles on the surface of CuS plates. Significant enhancement in photocatalytic activity of CuS was observed after surface modification by Au nanoparticle. CuS-Au-3 shows the best performance in photocatalytic decomposition of MB dye under visible light. On the other hand, Au nanoparticle modified CuS nanostructure function as an efficient electrocatalyst in electrocatalytic Hydrogen evolution reaction. Au nanoparticle present on the surface of CuS functioned as electron sink, which drags photo generated electron from CuS under irradiation of visible light and facilitate the MB degradation process nearly 21 times. Whereas, upon application of external potential Au nanoparticles helps in rapid charge transfer from CuS to electrolyte to enhance the electrocatalytic performance in Hydrogen evolution reaction.

## Methods

Materials: Cu(II)-sulfate hexahydrate, Thioacetamide (TAA) were purchased from Sisco Research laboratory. Ethanol, NaOH were purchased from Merck. HAuCl_4_ was purchased from Alfa-aesar. H_2_SO_4_, Nafion, Isopropanol were all of analytical grade and received from Sigma-Aldrich. Without any purification all chemicals were used.

### Synthesis of CuS nanostructure

Following a very simple wet-chemical route CuS was synthesized. Cu(II)-sulfate & TAA were used as precursor of Cu and S, respectively. 0.3 g of Cu(II)-sulfate was dissolved in 20 mL water and stirred for 5 min and labelled as solution A. On the other hand, 0.07 g of TAA was dissolved in 20 mL water and then 1 mL 1 M NaOH was added and sonicated for 10 min and marked as solution B. After that solution A was kept on a water bath at temperature ~80 °C. Then solution B was added drop wise to solution A. Finally, the whole mixture was kept on water bath for 30 min. the bluish-green color compound was collected by several washing with water and followed by ethanol.

### Decoration of Au nanoparticles on CuS nanostructure

The as-synthesized CuS nanostructure was used to synthesize Au nanoparticles where CuS function as reducing agent as well as support for the growth of Au nanoparticle. For this procedure, 30 mg of CuS nanostructure was thoroughly dispersed in 30 mL DI water via ultrasonication for 30 min. After that the dispersed CuS was kept under visible light irradiation in stirring condition. Different amounts of (1, 2, 3 and 4 mL 1.25 × 10^−2^ M HAuCl_4_) was added in the CuS dispersion and stirred for 1.5 h. 1 mL, 2 mL, 3 mL and 4 mL aliquots of HAuCl_4_ were added to CuS and named as CuS-Au-1 (5.92 weight % Au), CuS-Au-2 (12.85 weight % Au), CuS-Au-3 (19.98 weight % Au), and CuS-Au-4.

### Electrochemical Measurement

Electrochemical measurements were carried out in a three-electrode system. Aqueous solution of H_2_SO_4_ (10 mL of 0.5 M) was used as electrolyte. In this electrochemical experiment, Ag/AgCl, Pt-wire, and sample deposited on glassy carbon electrode (GCE) were used as reference electrode, counter electrode, and working electrode, respectively. All the electrochemical data was recorded in CH Instrument (CHI604E) at 25 °C. The linear-sweep voltammogram of CuS and CuS-Au-n (n = 1, 2, 3, 4) was obtained between 0.2 V to −0.8 V potential range applying 50 mV/S scan rate.

### Preparation of working electrode

Ink of CuS and CuS-Au-n were prepared by dispersing 5 mg of the sample in 300 μL of Isopropanol. Then 30 μL of nafion was added as binder and sonicated for 30 min for uniform dispersion. Finally, 5 μl dispersed compound was drop casted carefully on 3 mm diameter GC electrode.

### Electrochemical Impedance Spectroscopy

Electrochemical impedance measurement was also performed in a three electrode system. Onset potentials of different materials were chosen as the performing bias for this measurement with the sweeping of frequency from 50 KHz to 10 Hz with a 10 mV AC dither.

### Photocatalysis Study

To determine the photocatalytic activity of CuS and CuS-Au-3, Methylene blue (MB) was applied as a model dye. MB has a sharp optical absorption at 663 nm, which was used to monitor the degradation process and to study the kinetics. 10 mg of the desired catalyst was first immobilized in 30 mL of 2 × 10^−5^ M aqueous MB solution. After immobilization of catalyst molecule in the MB solution, it was stirred in dark for 30 min to establish the adsorption-desorption equilibrium. A tungsten bulb (100 W), which emits a continuous spectrum of light in between 300–1400 nm was used as visible light source to irradiate the solution after reaching to equilibrium. After agitation, the reaction mixture was kept under tungsten light to initiate the reaction with continuous stirring and the distance between the light source and reaction mixture was fixed at ~20 cm. Photocatalysis was carried out in neutral pH and room temperature. To monitor the photocatalysis process, at a regular interval 3.0 mL of aliquots were taken out from the reaction mixture and then centrifuged and used to check the optical absorbance. Remaining dye in the solution was quantified from the observed absorbance intensity. % Degradation was calculated from the given equation 4:


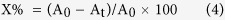


where, A_0_ = initial peak intensity and A_t_ = peak intensity at time ‘t’. A plot of ‘A_t_/A_0_’ vs.‘t’ was used to evaluate the order of the reaction and the corresponding ln(A_t_/A_0_) vs. ‘t’ was used to find out the rate constant of the reaction.

### Optical & Structural Characterization

Jasco V-650 Spectrophotometer (model no. UV-1800) with a deuterium and tungsten-halogen lamp was used to study ultra-violate-visible spectroscopy. Horiba Jobin Yvon Spectroflourimeter (Fluoro max-4) was used to measure the photoluminescence (PL) spectra. Rigaku Mini Flex II diffractometer with Cu-Kα radiation was utilized to monitor powder X-ray diffraction pattern, with a scanning rate 2° per min. Morphology of CuS and CuS-Au-n samples were investigated with the help Nova NanoSem 450 FESEM. Bruker XFlash 6130, attached with FESEM instrument was used for EDS analysis. Morphology of CuS and CuS-Au-3 sample was determined using Transmission electron microscopy (Bruker microscope operated). X-ray photoelectron spectroscopy (XPS) analysis was carried out using a commercial Omicron EA 125 source with Al-Kα radiation (1486.7 eV). Raman analysis was carried out using Airix (STR 500) instrument. ICP-OES analysis was carried out in Varian 720-ES.

### Mechanism for the decoration of Au Nanoparticle

**Figure 10:**
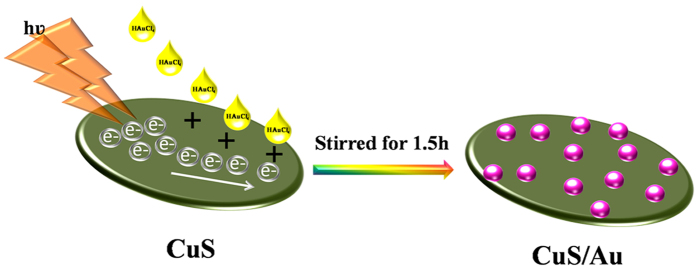
Schematic representation of the decoration of Au nanoparticles on CuS plates.

A green pathway (photochemical) was introduced for the synthesis of Au nanoparticle on the surface of CuS small plates. In this methodology, neither reducing agent nor any stabilizer was used. CuS is an excellent visible light active photocatalyst with a band gap of 2.2 eV 49. Upon irradiation of visible light there will be successive generation of electron (e−) and hole (h+) pair in the valence band (VB) and electrons will jump to conduction band (CB) with excessive energy. Added HAuCl_4_ undergoes photoreduction to Au(0) by these photogenerated electrons and deposited on the surface of CuS and represented in Fig. 10.

## Additional Information

**How to cite this article**: Basu, M. *et al.* Construction of CuS/Au Heterostructure through a Simple Photoreduction Route for Enhanced Electrochemical Hydrogen Evolution and Photocatalysis. *Sci. Rep.* **6**, 34738; doi: 10.1038/srep34738 (2016).

## Supplementary Material

Supplementary Information

## Figures and Tables

**Figure 1:**
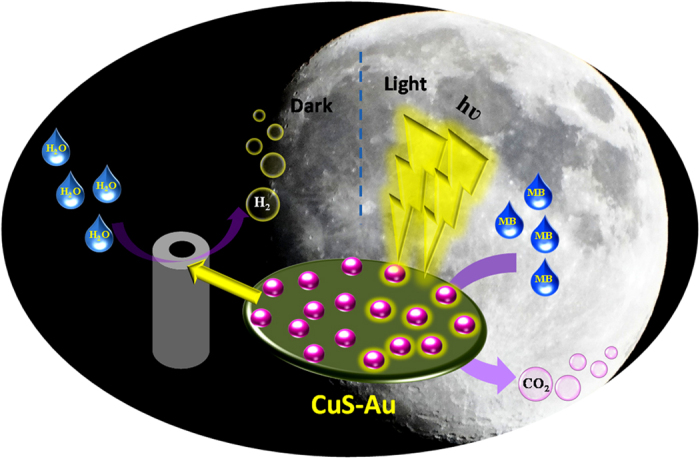
Schematic representation of the dual functionality of CuS/Au (photocatalysis under light irradiation and electrocatalytic activity under dark).

**Table 1:** Fitted Charge Transfer values of CuS, CuS-Au-2 and CuS-Au-3 on GC electrode.

Cathode	Rs(Ω)	Rct (Ω)
CuS	10.5	255.4
CuS-Au-2	11.7	143.7
CuS-Au-3	14.8	31.1
