# Supplementary material for: Construction of CuS/Au Heterostructure through a Simple Photoreduction Route for Enhanced Electrochemical Hydrogen Evolution and Photocatalysis
Source: Sci Rep. 2016 Oct 5;6:34738. doi: 10.1038/srep34738 (PMC5050419; doi:10.1038/srep34738)
Supplement: Supplementary Information [file srep34738-s1.pdf]

Supporting Information

Construction of CuS/Au Heterostructure through a Simple Photoreduction Route for Enhanced Electrochemical Hydrogen evolution and Photocatalysis

Mrinmoyee Basu,[*,a] Roshan Nazir,[a] Pragati Fageria[a] and Surojit Pande[*,a]

Department of Chemistry, BITS Pilani, Pilani, Rajasthan, 333031, India

Email: mrinmoyee.basu@gmail.com; surojitpande@gmail.com

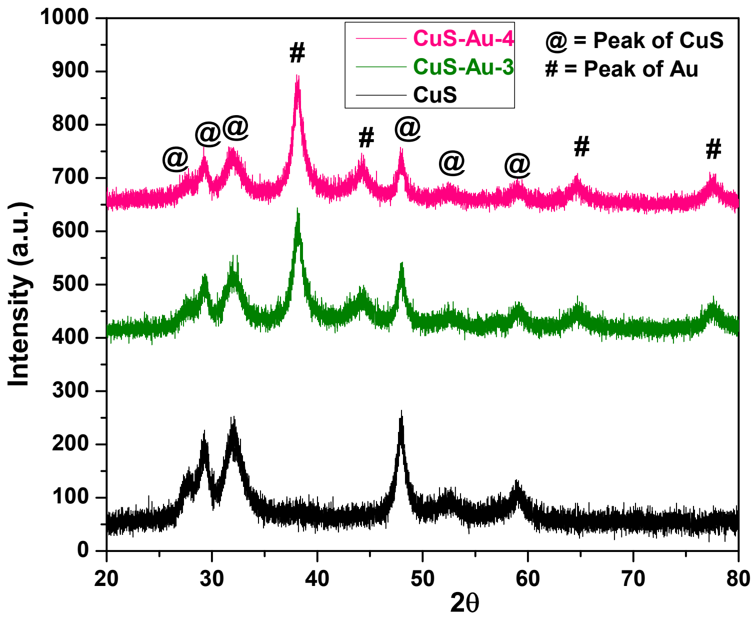

Figure S1: PXRD pattern of CuS and CuS-Au-n (n= 3, 4) showing the variation in the intensities of the highest intense peak of CuS and Au.

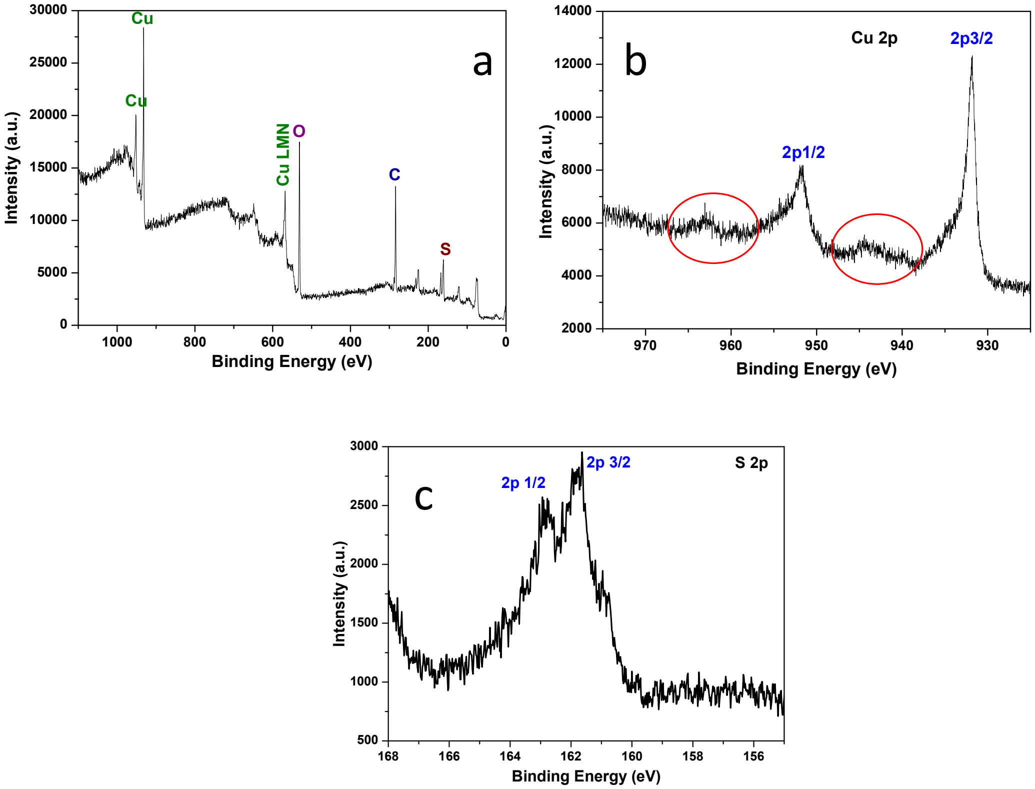

Figure S2: XPS spectra (a) wide scan spectra of CuS, (b) binding energy of Cu (2p) and (c) binding energy of S (2p) region.

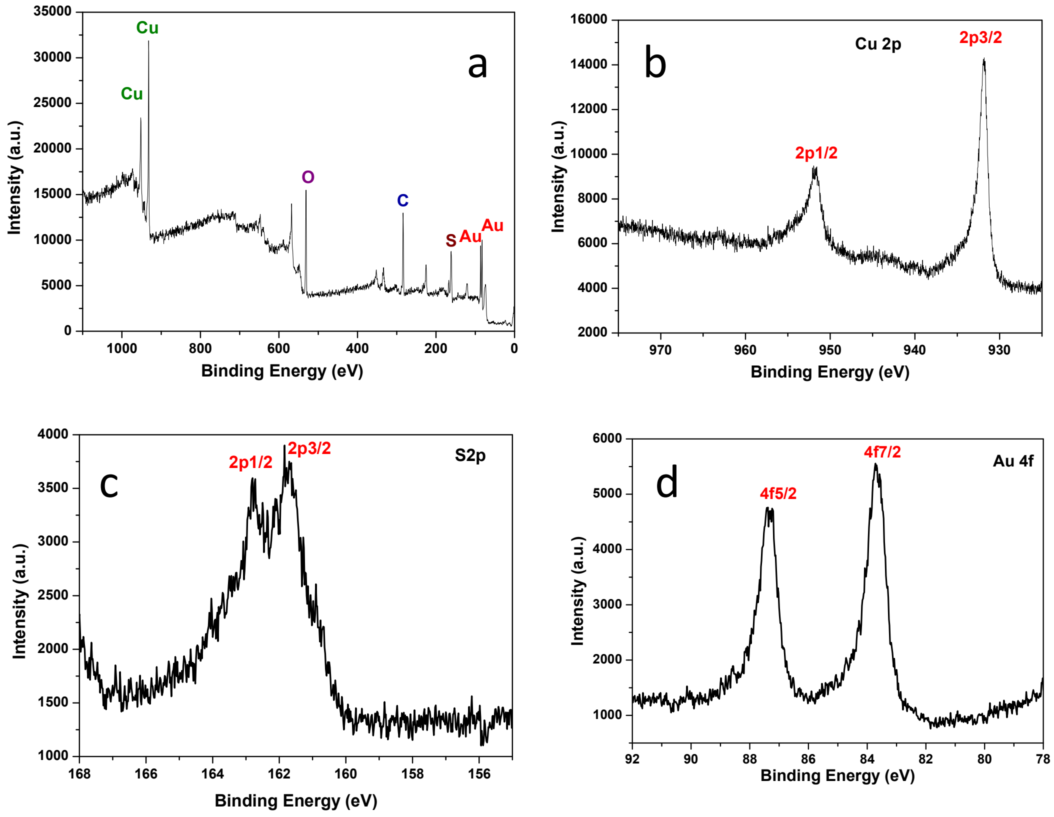

Figure S3: XPS spectra (a) wide scan spectra of CuS-Au-3, binding energy of (b) Cu (2p), (c) S (2p), and (d) Au (4f) region.

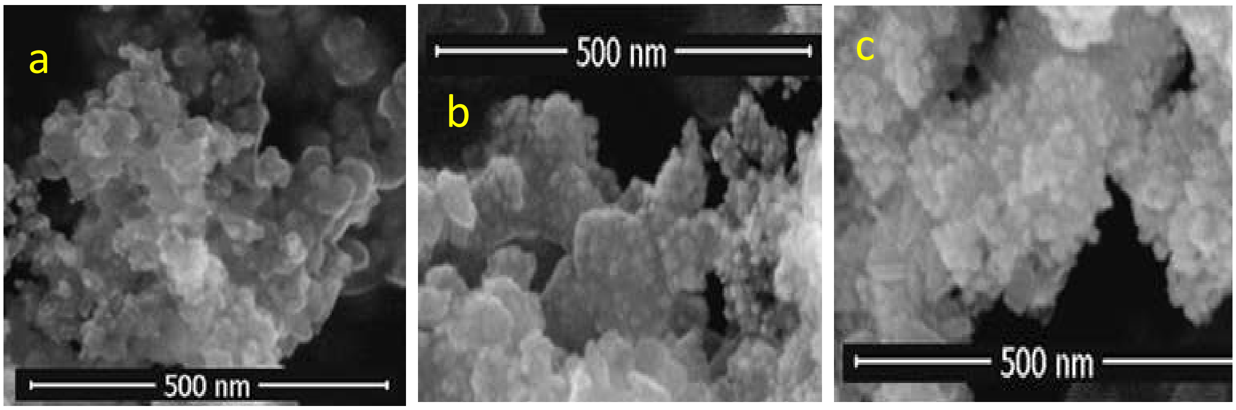

Figure S4: FESEM images of (a) CuS-Au-1, (b) CuS-Au-2, (c) CuS-Au-3 showing the presence of different Au loading on CuS surface.

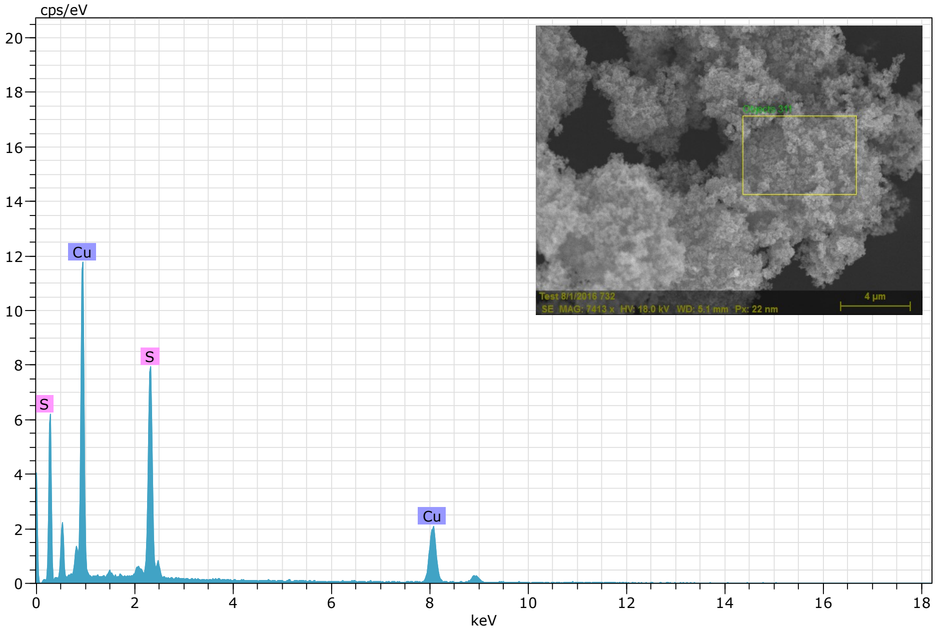

Figure S5: SEM-EDS of only CuS showing the presence of Cu and S.

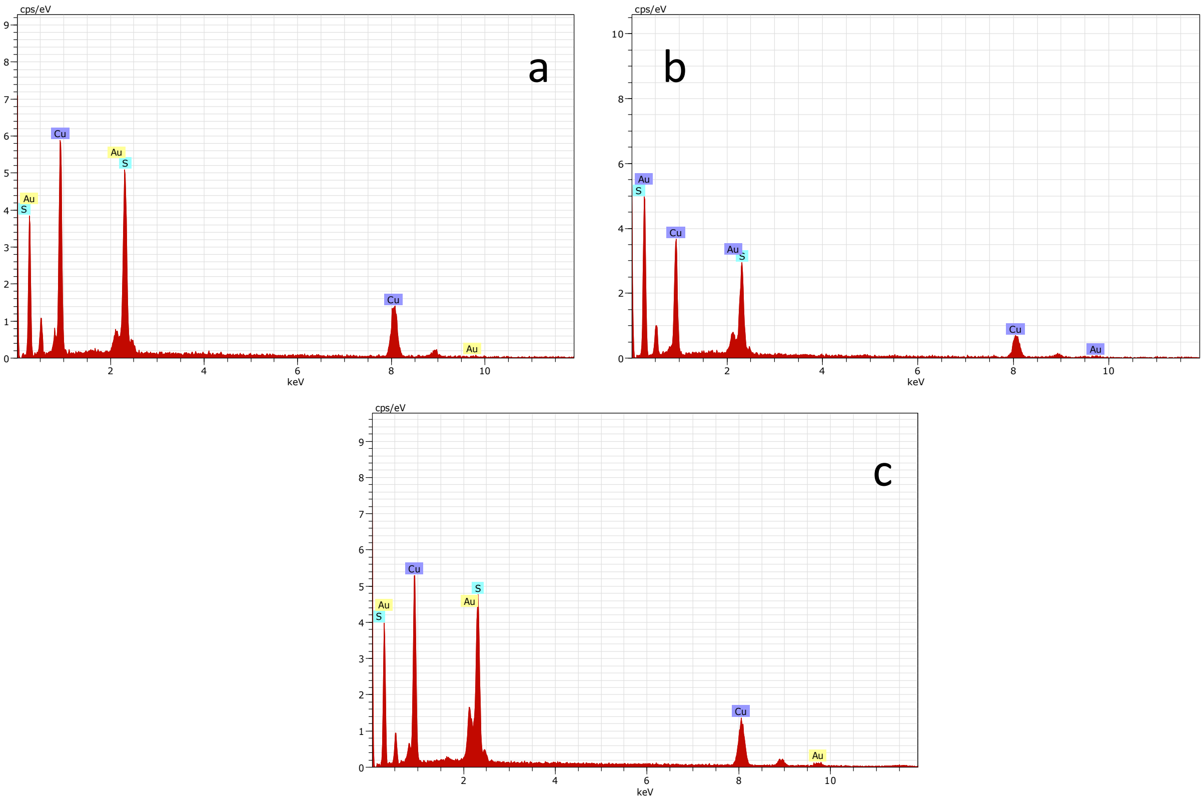

Figure S6: SEM-EDS analysis of (a) CuS-Au-1, (b) CuS-Au-2, and (c) CuS-Au-3

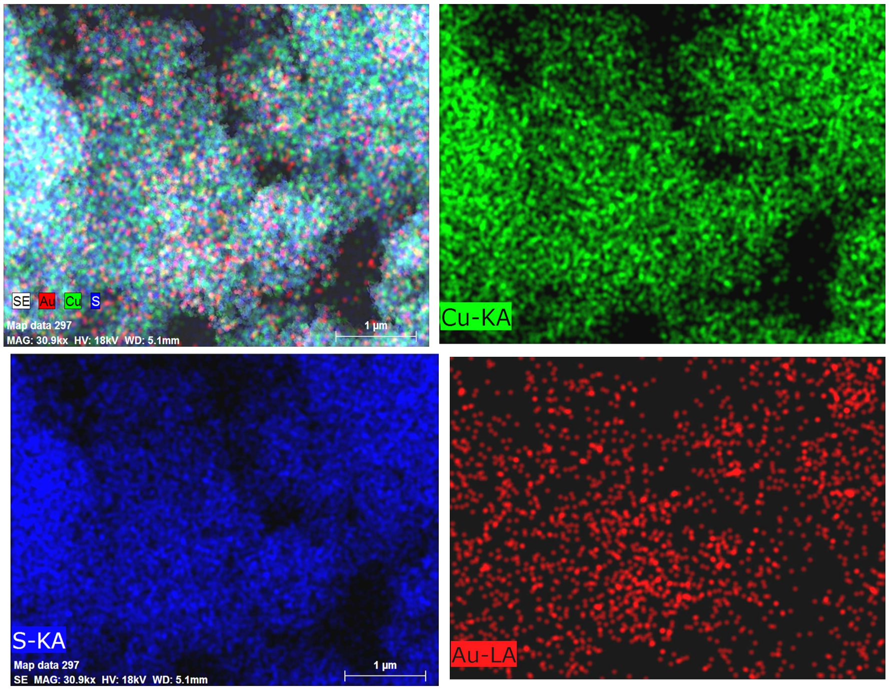

Figure S7: SEM-EDS mapping of CuS-Au-3 showing the presence of Cu, Au, and S.

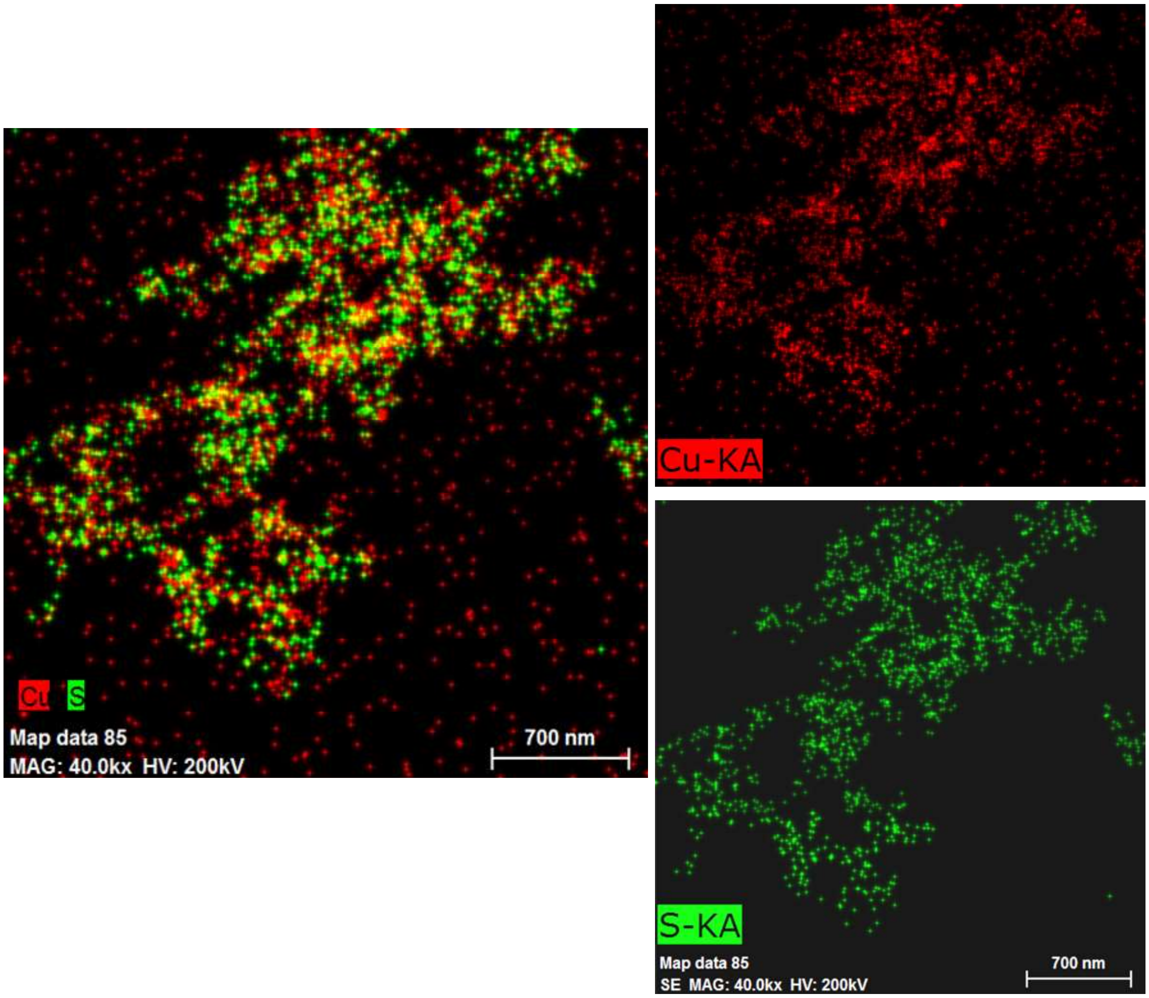

Figure S8: TEM-EDS mapping of only CuS showing the presence of Cu and S.

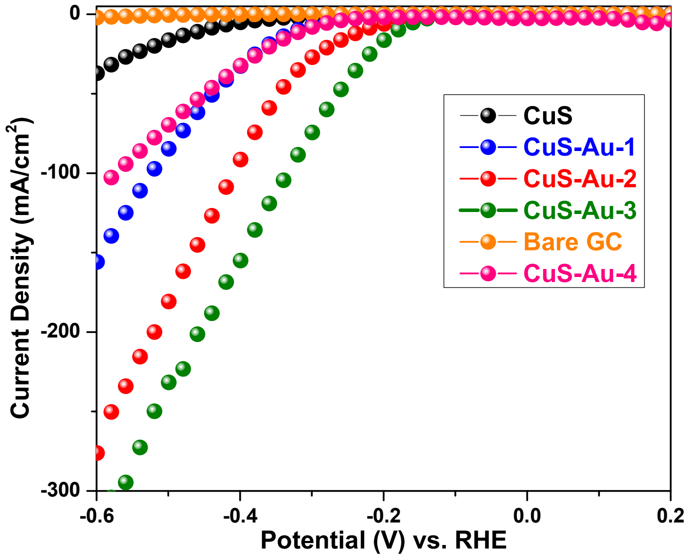

Figure S9: Polarization curves for bare GC, CuS, CuS-Au-1, CuS-Au-2, CuS-Au-3, and CuS-Au-4 in 0.5 M H_2SO_4.

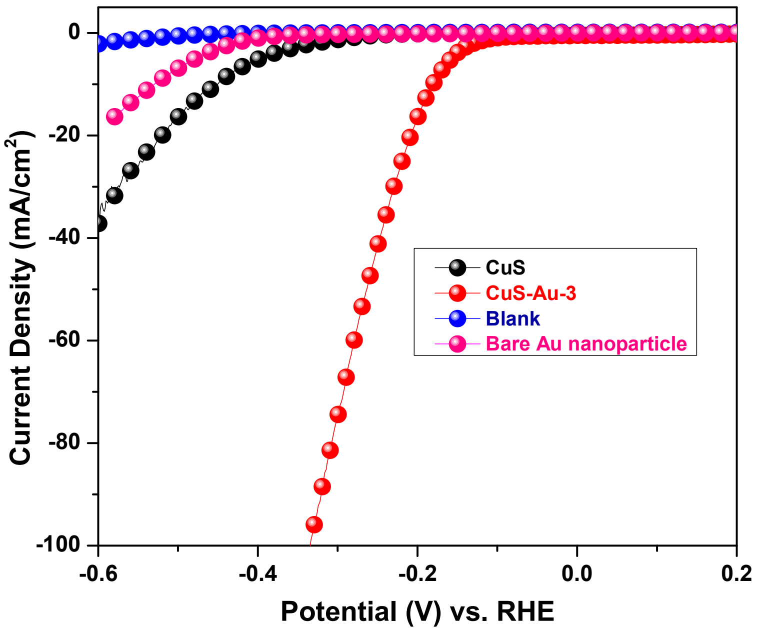

Figure S10: Polarization curves for bare GC, pure Au nanoparticle, CuS, and CuS-Au-3 in 0.5 M H_2SO_4.

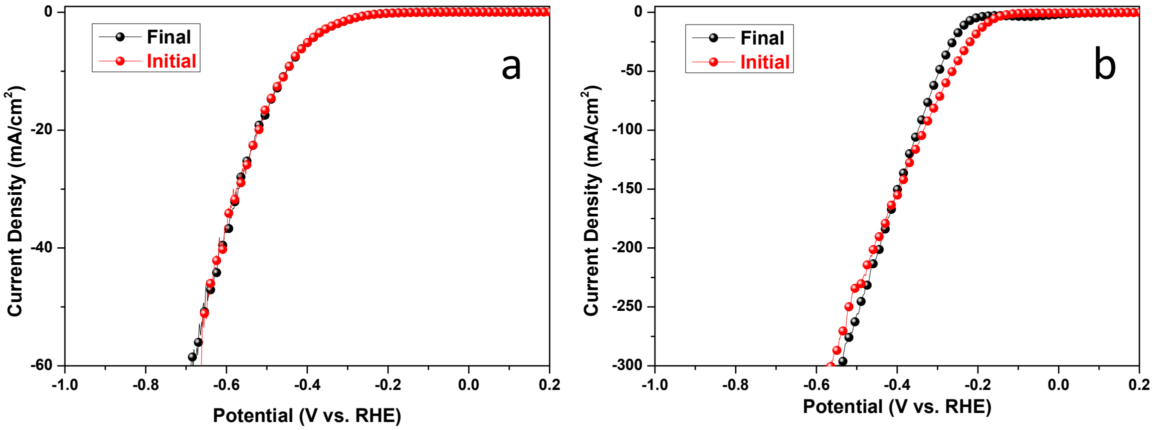

Figure S11: Comparative polarization curve of (a) CuS and (b) CuS-Au-3, initial run and after 1000 cycle.

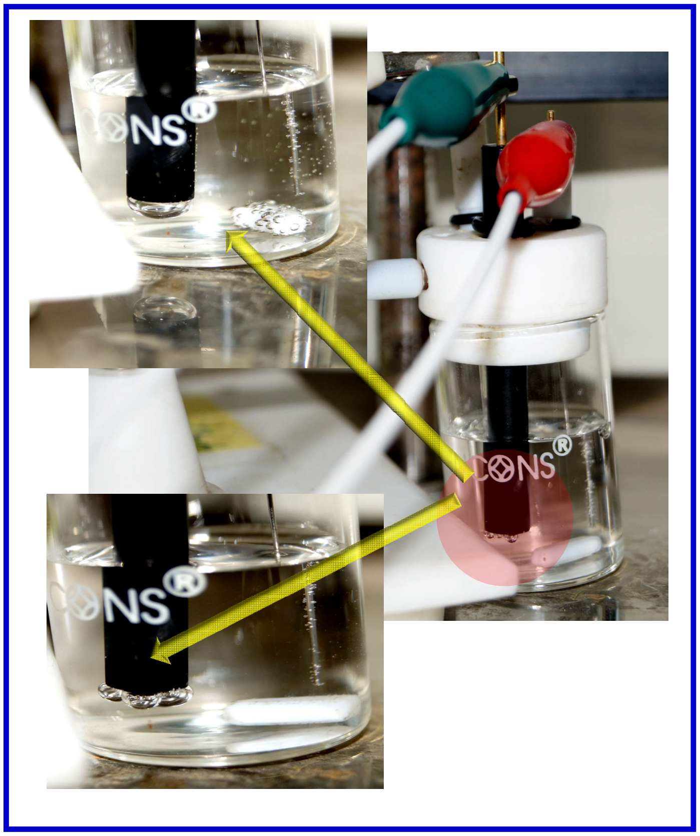

Figure S12: Digital image of hydrogen evolution in case of CuS-Au-3.

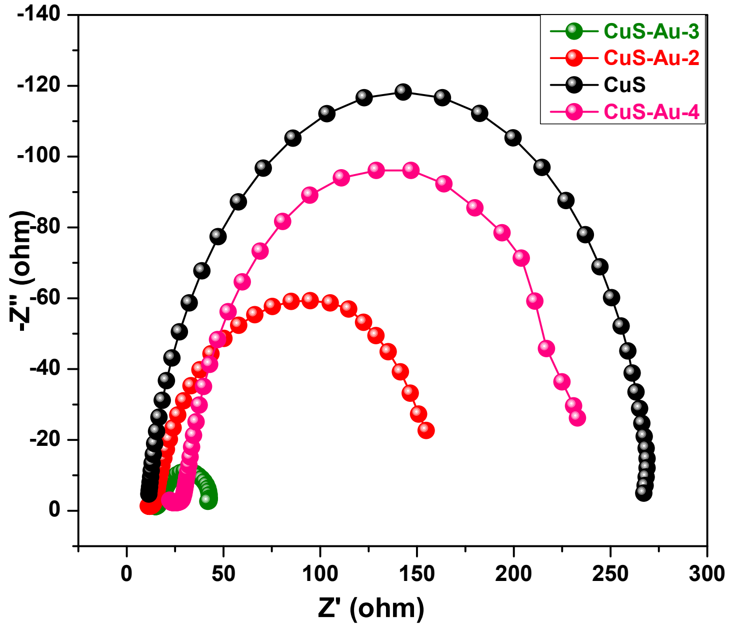

Figure S13: Comparative Nyquist plot of CuS, CuS-Au-2, CuS-Au-3, and CuS-Au-4.

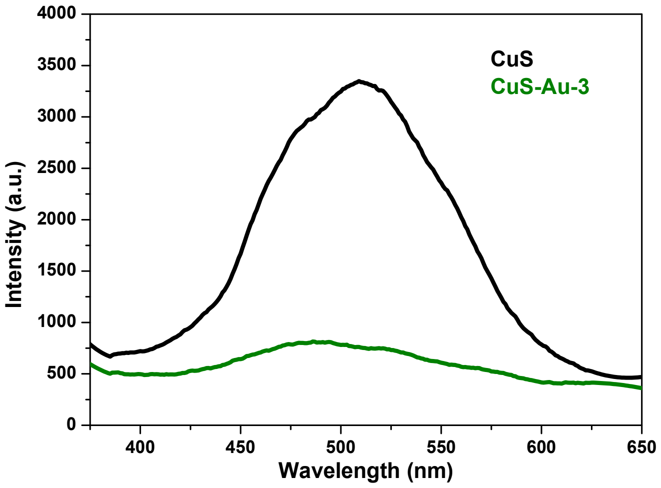

Figure S14: Comparative PL spectra of CuS and CuS-Au-3.

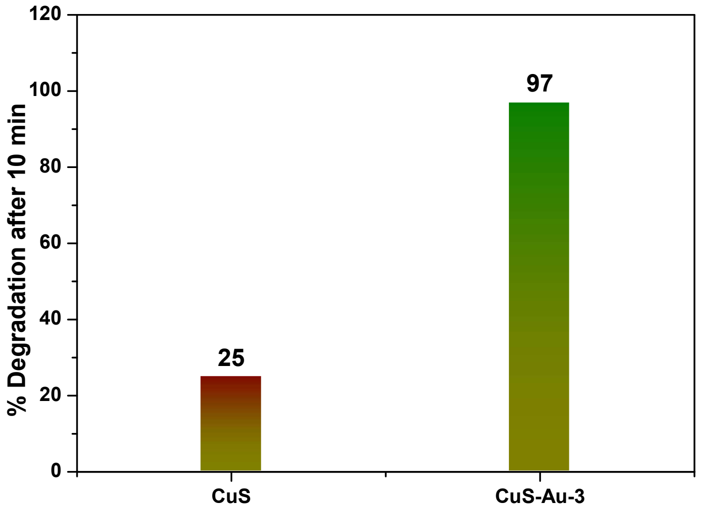

Figure S15: Comparative photocatalytic study of CuS and CuS-Au-3 showing the bar diagram of % dye removal efficiency.

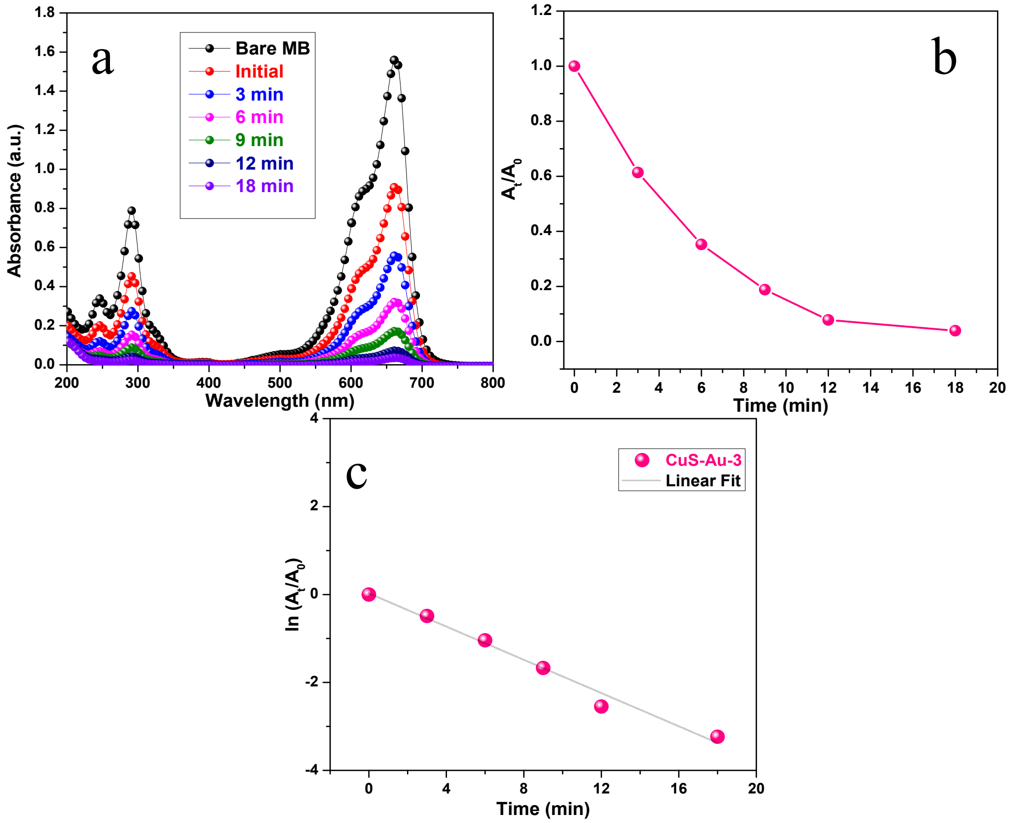

Figure S16: Photocatalytic decomposition of MB dye by using CuS-Au-3 (a) absorbance vs. wavelength plot, (b) Plot of A_t/A_0 vs. time shows the kinetics and (d) Plot of ln (A_t/A_0) vs. time. Conditions, [MB] = 2.5 x 10^{-5} M and catalyst = 10 mg.

Sample ID	UOM	Gold as Au
CuS/Au-1	% by mass	5.92
CuS/Au-2	% by mass	12.85
CuS/Au-3	% by mass	19.98

Table S1: ICP-OES data for CuS-Au-n (n= 1, 2, 3).
